# Center of Pressure Density Maps as Postural Asymmetry Indicators and Biofeedback for Older Adults

**DOI:** 10.1093/geroni/igaf122.3436

**Published:** 2025-12-31

**Authors:** Jethro Raphael Suarez, Camilla Torres, Joon-Hyuk Park, Ladda Thiamwong

**Affiliations:** University of Central Florida, Florida, United States; University of Central Florida, Orlando, Florida, United States; University of Central Florida, Orlando, Florida, United States; University of Central Florida, Orlando, Florida, United States

## Abstract

Nonvisual balance tests enhance proprioceptive and vestibular system use to maintain balance due to the absence of visual input. Visual tools exist that show center of pressure (COP) movement during such tests, but many are difficult to understand visually and do not give focus to the initial starting point. This study aimed to develop density maps from raw force plate data for use as a form of biofeedback for community-dwelling older adults. The Balance Tracking System (BTrackS) is a simple, cost-effective force plate system that records COP movement and was used for balance assessments. R software was utilized to generate density maps using the “stat_density2d” function in the “ggplot2” library from the raw data produced by the BTrackS. We collected force plate data from 21 community-dwelling older adults aged 64 to 83 (mean age = 72.3 ± 5.9 years) who completed a static balance test on the BTrackS. The static balance test involved standing still with feet shoulder-width apart, hands on hips, head forward, and eyes closed for four 20 second trials with 10 seconds rest between. Density maps revealed that the highest COP density areas were not located near the initial starting point for a few older adults, indicating postural asymmetry. The results show that COP density maps could serve as beneficial feedback for older adults by allowing for the recognition of postural sway asymmetry. Future research should determine the feasibility of providing such feedback for older adults, as well as investigate factors that could cause such asymmetry.

